# Omental Infarction Mimicking Acute Appendicitis: A Case Report

**DOI:** 10.7759/cureus.47232

**Published:** 2023-10-17

**Authors:** Hadi S Alyami, Saleh M Almasaabi, Hadi A Al Swaidan, Hassan Dhaen

**Affiliations:** 1 College of Medicine, King Faisal University, Hofuf, SAU; 2 Emergency Medicine, King Khalid Hospital, Najran, SAU; 3 College of Medicine, Arabian Gulf University, Manama, BHR

**Keywords:** case report, computed tomography, omental infarction, appendicitis, acute abdomen

## Abstract

Acute abdominal pain is a common presentation in emergency departments, often attributed to a myriad of potential causes. Among these, acute appendicitis remains a frequently diagnosed culprit. However, this case report presents a 32-year-old male who arrived at the emergency department with severe right lower quadrant abdominal pain, characterized by localized tenderness and guarding. The initial clinical diagnosis pointed to acute appendicitis. Before surgical intervention, a computed tomography scan was conducted and revealed a focal area of fat stranding, consistent with omental infarction, while the appendix appeared normal. The patient's management involved pain control and supportive care, leading to a complete resolution of abdominal pain at a two-week follow-up. This case emphasizes the significance of including omental infarction in the spectrum of diagnoses for acute abdominal pain, underlining the potential to prevent unnecessary surgical interventions.

## Introduction

Acute abdominal pain is a prevalent complaint among patients presenting to emergency departments, accounting for approximately 10% of all emergency visits [1]. The differential diagnosis of acute abdominal pain is complex, as many conditions can present with similar symptoms, including gastrointestinal, genitourinary, gynecological, and vascular causes, as well as non-abdominal causes such as pulmonary, cardiac, or musculoskeletal disorders [1,2].

This case report aims to shed light on a less commonly encountered but important condition in the context of acute abdominal pain: omental infarction [3]. While appendicitis is a well-known and frequently diagnosed cause of such pain, omental infarction, characterized by the occlusion of the omental blood supply leading to ischemia and necrosis, can mimic the clinical presentation of appendicitis [3]. Omental infarction is often under-recognized, and this report highlights the significance of considering it in the differential diagnosis. We present a rare case in which omental infarction masquerades as acute appendicitis, underscoring the importance of awareness and consideration of this condition when evaluating patients with acute abdominal pain.

## Case presentation

We present the case of a 32-year-old male who sought medical attention in the emergency department due to severe right lower quadrant abdominal pain persisting for 24 hours. The patient described the pain as sharp, constant, and progressively worsening. He had a history of well-controlled hypertension managed with captopril and had no significant prior surgeries or allergies to food or medications. He neither smoked nor consumed alcohol or drugs.

Upon physical examination, the patient appeared uncomfortable, with a body temperature of 99.8 °F (37.7 °C), a heart rate of 96 beats per minute, and a blood pressure of 132/82 mmHg. Tenderness and guarding were noted upon abdominal palpation in the right lower quadrant, but there was no palpable mass, and bowel sounds were present.

Laboratory tests revealed a high white blood cell count of 13.4 × 109/L with 85% neutrophils, while C-reactive protein and erythrocyte sedimentation rate levels were within the normal range. The patient's hemoglobin level was 13.5 g/dL, and serum electrolyte and liver function panels showed no abnormalities. Urinalysis returned normal results.

**Figure 1:**
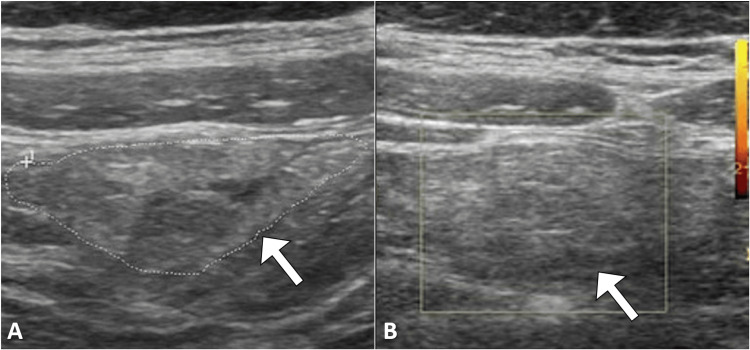
Gray-scale ultrasound image (A) shows a focal area of omental fat with increased echogenicity (arrow), while the corresponding color Doppler image (B) reveals no blood flow.

An initial abdominal ultrasound did not visualize the appendix but did reveal a focal area of increased echogenicity in the omental fat within the right lower quadrant, corresponding to the patient's maximal tenderness point (Figure 1). No gallbladder or renal pathology was evident. Given the equivocal findings, a computed tomography scan was recommended.

**Figure 2:**
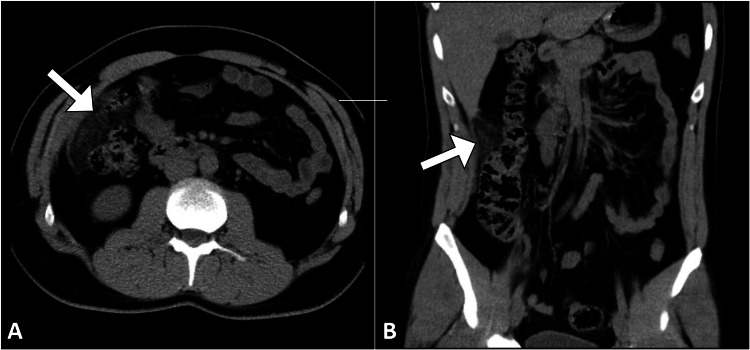
Axial (A) and coronal (B) CT images display a localized focal area of increased fat attenuation within the omentum (arrow), consistent with acute omental infarction. CT: computed tomography.

Subsequently, the patient underwent a computed tomography scan, which identified a focal area of fat stranding in the greater omentum with a central region of hypoattenuation in the right upper quadrant infra-hepatic area, accompanied by peritoneal thickening (Figure 2). Importantly, the appendix appeared normal, with no evidence of acute appendicitis. These findings were consistent with a diagnosis of omental infarction.

Following the diagnosis of omental infarction, the patient was managed conservatively with pain control and supportive care. Over the next few days, the patient's pain gradually improved, and he could tolerate oral intake. He was discharged with instructions to continue pain control medication and to follow up with his primary care physician. During the two-week follow-up appointment, the patient reported complete resolution of his abdominal pain. Given the improvement in symptoms, a repeat computed tomography scan was not deemed necessary.

## Discussion

Acute omental infarction is an infrequent condition resulting from the occlusion of the omental blood supply, leading to ischemia and necrosis. While the exact etiology of omental infarction remains unclear, it is commonly associated with torsion, thrombosis, or embolism of the omental vessels [3]. Torsion, wherein the omentum twists around its vascular pedicle, is believed to be the most prevalent cause of omental infarction, accounting for approximately 60-80% of cases [3,4].

The clinical presentation of omental infarction can mimic that of acute appendicitis. Patients often experience sudden-onset, severe abdominal pain localized to the right or left lower quadrant, accompanied by symptoms such as nausea, vomiting, and fever [4]. Physical examination may reveal localized tenderness and rebound tenderness. However, in contrast to acute appendicitis, patients with omental infarction may not exhibit associated anorexia, bowel changes, or other signs of peritoneal irritation [3].

Radiological imaging, including ultrasound or computed tomography, can assist in diagnosing omental infarction by identifying a well-defined, hyperechoic, or hyperdense mass in the omentum, with or without surrounding fluid [3,5]. Nevertheless, the diagnosis is often made intraoperatively due to the nonspecific clinical presentation and imaging findings [3].

The management of omental infarction typically involves a conservative approach with antibiotics, analgesics, and rest [3]. Conservative management aims to control pain, manage fever, and prevent secondary infections. The duration of antibiotic therapy depends on the severity of the infarction and the presence of associated complications. In most instances, symptoms of omental infarction resolve within a few days to weeks with conservative management alone [4,5].

Surgical intervention may become necessary in certain situations, such as diagnostic uncertainty, failure of conservative management, or the presence of complications like abscess formation or peritonitis. The choice of surgical approach depends on the extent of the infarction and the presence of complications. Laparoscopic omentectomy may be performed in some cases, while others may necessitate more extensive surgical resection [3,6].

## Conclusions

Omental infarction is a relatively uncommon cause of acute abdominal pain that can mimic other, more common causes, such as acute appendicitis. Emergency physicians should be aware of the possibility of omental infarction and consider imaging as necessary to confirm the diagnosis. Conservative management, including pain control and supportive care, is typically sufficient for symptom resolution, and surgical intervention is seldom required but may be necessary in rare cases.
